# Dynamic Functional Connectivity and Symptoms of Parkinson’s Disease: A Resting-State fMRI Study

**DOI:** 10.3389/fnagi.2018.00388

**Published:** 2018-11-23

**Authors:** Gwenda Engels, Annemarie Vlaar, Brónagh McCoy, Erik Scherder, Linda Douw

**Affiliations:** ^1^Department of Clinical, Neuro and Developmental Psychology, Faculty of Behavior and Movement Sciences, VU University, Amsterdam, Netherlands; ^2^Department of Neurology, Onze Lieve Vrouwe Gasthuis (OLVG), Amsterdam, Netherlands; ^3^Department of Experimental and Applied Psychology & Institute of Brain and Behavior, Faculty of Behavior and Movement Sciences, VU University, Amsterdam, Netherlands; ^4^Department of Anatomy and Neurosciences, VU University Medical Center, Amsterdam, Netherlands; ^5^Department of Radiology, Athinoula A. Martinos Center for Biomedical Imaging, Massachusetts General Hospital, Charlestown, MA, United States

**Keywords:** dynamic functional connectivity, default mode network, frontoparietal network, Parkinson’s disease, cognitive impairment, pain, motor symptoms

## Abstract

Research has shown that dynamic functional connectivity (dFC) in Parkinson’s disease (PD) is associated with better attention performance and with motor symptom severity. In the current study, we aimed to investigate dFC of both the default mode network (DMN) and the frontoparietal network (FPN) as neural correlates of cognitive functioning in patients with PD. Additionally, we investigated pain and motor problems as symptoms of PD in relation to dFC. Twenty-four PD patients and 27 healthy controls participated in this study. Memory and executive functioning were assessed with neuropsychological tests. Pain was assessed with the Numeric Rating Scale (NRS); motor symptom severity was assessed with the Unified Parkinson’s Disease Rating Scale (UPDRS). All subjects underwent resting-state functional magnetic resonance imaging (fMRI), from which dFC was defined by calculating the variability of functional connectivity over a number of sliding windows within each scan. dFC of both the DMN and FPN with the rest of the brain was calculated. Patients performed worse on tests of visuospatial memory, verbal memory and working memory. No difference existed between groups regarding dFC of the DMN nor the FPN with the rest of the brain. A positive correlation existed between dFC of the DMN and visuospatial memory. Our results suggest that dynamics during the resting state are a neural correlate of visuospatial memory in PD patients. Furthermore, we suggest that brain dynamics of the DMN, as measured with dFC, could be a phenomenon specifically linked to cognitive functioning in PD, but not to other symptoms.

## Introduction

The organization of structural and functional connections of the brain, or brain topology, shapes complex behavior (Park and Friston, 2013; Stam, 2014): cognitive functioning arises from both local and global integration across the whole brain. Local integration, which mainly entails short-range neural connections, subserves specialized cognitive functions. Global integration entails long-range neural connections, and underlies higher cognitive functions (Park and Friston, 2013; Sporns, 2013). Cognitive dysfunctioning has been related to altered brain network topology as measured by resting-state imaging in several disorders, such as in multiple sclerosis (Hawellek et al., 2011; Meijer et al., 2017), schizophrenia (Lynall et al., 2010) and epilepsy (Douw et al., 2011).

Parkinson’s disease (PD) is a neurodegenerative disorder, hallmarked by motor symptoms: rigidity, bradykinesia, tremor and postural instability. Although clinical diagnosis of PD is based on these motor symptoms, non-motor symptoms play an equally, sometimes even more devastating role in patients’ quality of life (Martinez-Martin et al., 2011). One such non-motor symptom is cognitive decline, e.g., in the executive and memory domains (Litvan et al., 2012). In line with the abovementioned link between network integration and cognition, cognitive decline in PD has been associated with decreased functional connectivity (Olde Dubbelink et al., 2014; Amboni et al., 2015) or deviant coupling between subnetworks of the brain (Putcha et al., 2016). Another non-motor symptom is pain (Broen et al., 2012; Choi et al., 2017), a symptom that is present in about two thirds of patients and has been associated with sleep and mood disturbances, as well as severity of motor symptoms (Broen et al., 2012; Defazio et al., 2017). Pain in PD has been linked to a functional disconnection between the hippocampus and nucleus accumbens (Polli et al., 2016).

Connectivity analyses on resting-state functional magnetic resonance imaging (rs-fMRI) have, until recently, been based on the assumption that connections remain stable throughout an fMRI session. However, temporal fluctuations of connectivity may in fact be a fundamental feature of brain networks, particularly in the context of cognitive functioning (Sizemore and Bassett, 2017). Dynamic functional connectivity (dFC) is an approach to assess these temporal fluctuations, by calculating the variability of functional connectivity over a number of sliding windows within each scan. dFC may have added value in explaining cognitive functioning above and beyond that of static connectivity, as it may detect the brain network’s ability to deal with varying demands from the environment. Higher dFC (throughout the brain) has been related to better performance in healthy controls in the domains of cognitive flexibility, sustained attention and working memory (Jia et al., 2014). Also, lower dFC of a hub region of the default mode network (DMN), the posterior cingulate cortex (PCC), was associated with worse memory performance in epilepsy patients, suggesting that high dFC is favorable in the memory domain (Douw et al., 2015).

The DMN is a task-negative network (i.e., deactivated during cognitive processes) that appears to be particularly vulnerable to the effects of disease (Broyd et al., 2009). Dynamics of the DMN appear to be associated with internally-oriented cognition (Kucyi and Davis, 2014). Another network that is involved in cognitive functioning is the frontoparietal network (FPN), which plays a central role in flexibly adapting to the environment (Cole et al., 2013). Dynamics of the FPN have been implicated in cognitive control (Zanto and Gazzaley, 2013). In healthy controls, a lower resting-state dFC between FPN and the DMN was related to a higher cognitive flexibility (Douw et al., 2016). Regarding cognitive functioning in PD, a higher dFC of the dorsal attentional network at rest appears to be beneficial for performance on an attention task (Madhyastha et al., 2015a,b). Although dFC has been suggested to be associated with mild cognitive impairment (MCI) before (Jones et al., 2012), specific cognitive domains have not yet been investigated with respect to dFC in PD.

The relationship between dFC and other symptoms of PD, such as pain or motor symptom severity, has been largely underexplored. One study investigated dynamics of the resting state in PD by determining brain states with a sliding window technique. Two brain states were found: one relatively more integrated and one more segregated brain state. Time spent in the more integrated brain state was correlated with the severity of motor symptoms of PD patients (Kim et al., 2017). Pain in general, i.e., not specifically related to PD, may also depend on dynamic properties of the brain (Kucyi et al., 2013; Kucyi and Davis, 2015). However, this association seems primarily related to the modulation of pain by the cognitive domain of attention, instead of pain itself: a higher tendency to attend to painful stimuli was related to higher dFC.

This study investigates behavioral correlates of brain dynamics during the resting-state in PD. The primary aim is to investigate whether dFC of the DMN and the FPN is a correlate of cognitive functioning. More specifically, we hypothesize that dFC of the DMN with the rest of the brain shows an association with memory functioning and that dFC of the FPN with the rest of the brain shows an association with executive functioning. We will further investigate dFC in PD by assessing a possible difference between PD patients with MCI and those who have intact cognitive functioning. In addition to cognition, we will investigate whether dFC relates to motor problems and pain in PD patients.

## Materials and Methods

### Subjects

Patients were referred through neurologists of outpatient clinics (VU University Medical Center, Amsterdam, Netherlands; OLVG Hospital, Amsterdam, Netherlands; Zaans Medical Center, Zaandam, Netherlands). Healthy controls were recruited through advertisement in local newspapers, online advertisement and through participating patients (e.g., spouses, relatives, etc.). Inclusion criteria were: (1) age 40–75 years old; (2) ability to provide written informed consent; (3) normal or corrected-to-normal vision; and (4) for patients only, a diagnosis of PD following UK Brain Bank criteria. Exclusion criteria for all subjects were: (1) current use of psychotropic medication other than levodopa, dopamine-agonists or other Parkinson medication; (2) major somatic disorder; (3) current psychiatric diagnosis as established by a psychiatrist and (4) presence of dementia, history of stroke or other neurological diseases (as stated in their medical status). A screening for dementia was performed using the Montreal Cognitive Assessment (MoCA; Nasreddine et al., 2005), with a cutoff of 21 or lower for dementia according to Biundo et al. (2014). Additionally, Beck’s Depression Inventory (BDI) was administered to assess presence of depressive symptoms (Beck et al., 1996). Patients and controls were matched for age and sex. Education was categorized according to the Verhage-system, which runs from 1 (unfinished lower education) to 7 (finished university level; Verhage, 1964).

The study was carried out in accordance with regulations of the medical ethical committee of the VU University Medical Center (Amsterdam, Netherlands), who also approved the protocol. All subjects gave written informed consent in accordance with the Declaration of Helsinki. All methods were carried out in accordance with relevant guidelines and regulations.

### Procedure

This project was part of a larger study investigating reinforcement learning, visual attention, and pain in PD. Sample sizes of 24 patients and 24 controls were pre-determined for the reinforcement learning task, however for the current study we could include additional subjects who passed the initial screening procedure (see “Results” section). Patients visited the hospital three times and controls twice. On the first day, all subjects underwent clinical assessment including neuropsychological testing, practiced a reward-learning task and filled out questionnaires.

Patients underwent MRI twice: once with their normal Parkinson medication (ON phase), once without Parkinson medication (OFF phase). Only OFF phase imaging was used for this study in order to reduce the effect of dopaminergic medication. The OFF phase was defined as at least 12 h of dopaminergic medication withdrawal (overnight). One patient took their medication 8.5 h before the resting-state scan to relieve motor symptoms. Controls underwent MRI once. Note that only those procedures concerning the current project will be described in the remainder of the text. The MRI was generally planned in the same week as the clinical assessment, but no later than 60 days after clinical assessment.

### Clinical Assessment

#### Cognition, Motor Symptoms and Pain

Since neuropsychological testing was performed on a different day than the scanning day, patients took their medication as normal, thus cognition was not tested during the OFF phase.

##### Executive Functioning

The Stroop Color Word test was used to investigate interference (Stroop, 1935). The interference measure was calculated by dividing the time needed for Stroop, card II (colored rectangles) by time needed for Stroop, card III (names of colors written in incongruent colors; Lansbergen et al., 2007), with a high score indicating a low degree of interference. To test verbal fluency, subjects were asked to generate as many words as possible from a specific semantic category within 1 min, which was repeated with a different category (categories were first animals, then professions). Subjects were then asked to generate as many words as possible starting with a specific letter, which was repeated twice (letters were D, A and T). Word fluency was the average of both the category and the letter fluency tests. The Digit Span Backwards from the Wechsler Adult Intelligence Scale was administered to test working memory (Wechsler, 1955). Subjects were asked to repeat a series of digits in a reversed order. The number of correctly repeated series was used as working memory measure. The Rule-Shift Cards Test of the Behavioral Assessment of the Dysexecutive Syndrome (BADS) was used to assess mental flexibility (Wilson et al., 1998). Subjects were asked to respond to a set of stimulus cards according to a rule. In the second condition, the rule was changed. Number of mistakes was used to calculate a profile score, which served as a measure for mental flexibility.

##### Memory

Verbal memory was assessed using the Rey Auditory Verbal Learning Test (AVLT). The Dutch version of this test encompasses a list of 15 unrelated words (Saan and Deelman, 1986). In the immediate recall condition, the total number of correct words after five trials served as a measure for short-term verbal memory. In the delayed recall condition, the number of correct words after an interval of at least 15 min was used as a measure for long-term verbal memory. The Complex Figure of Rey (CFR) was used as an indication of visuospatial memory. Subjects were asked to copy a complex figure. After several minutes, subjects were asked to reproduce the figure without the original (Osterrieth, 1941). Scoring was performed according to a standardized method.

##### Motor Functioning

The motor part of the Unified Parkinson’s Disease Rating Scale (UPDRS III) was administered during patients’ OFF phase before MRI (Postuma et al., 2015). UPDRS III was always performed by the same person (GE).

##### Pain Experience

The Numeric Rating Scale (NRS) was administered before commencing the resting-state scan during the OFF phase. Subjects were asked to rate their pain on a scale from 0–10 for intensity of pain, with 0 indicating “no pain,” and 10 indicating “the worst pain ever experienced.”

### MRI

Imaging data were collected with a 3T GE Signa HDxT MRI scanner (General Electric, Milwaukee, WI, USA) at the VU University Medical Center (Amsterdam, Netherlands). Structural images were acquired with a 3D T1-weighted magnetization prepared rapid gradient echo (MPRAGE) sequence with the following acquisition parameters: voxel size = 1 mm isotropic, 176 slices, 256 × 256 matrix, repetition time (TR) = 8.2 ms, echo time (TE) = 3.2 ms, flip angle (FA) = 12 degrees, inversion time (TI) = 450 ms. Resting-state data were acquired using a T2*-weighted echo-planar functional scan: number of volumes = 202, 42 slices, slice thickness = 3.2 mm, matrix size = 64 × 64, TR = 2150 ms, TE = 35 ms, FA = 80 degrees, field of view (FOV) = 240 mm, total duration 7:12 min. For the resting-state scan, subjects were instructed to close their eyes, lie still and avoid falling asleep. The subject’s head was immobilized using foam pads to reduce motion artifacts.

#### Processing of fMRI Data

Data were analyzed using FSL FMRIB software library v5.0.9 (Jenkinson et al., 2012) and custom built scripts in bash and Matlab, version 2015a (Mathworks, Natick, MA, USA). The following pre-processing steps were taken: (1) images were corrected for head motion using MCFLIRT (Jenkinson et al., 2012); (2) bottom-up slice-timing correction was applied; (3) non-brain tissue was removed (using Brain Extraction Tool, BET); (4) functional images were registered to subject-space (T1-weighted structural image) using BBR; (5) the T1-weighted structural image was registered to MNI152 standard space (FLIRT for linear registration with 12 DOF); (6) high-pass filtering above 0.01 Hz was applied; (7) spatial smoothing was performed at 5 mm full-width half maximum (FWHM); (8) segmentation of gray and white matter was performed using FAST and SIENAX; and (9) the first three volumes of each resting-state scan were discarded to achieve field equilibrium.

#### Dynamic Functional Connectivity

**Figure 1:**
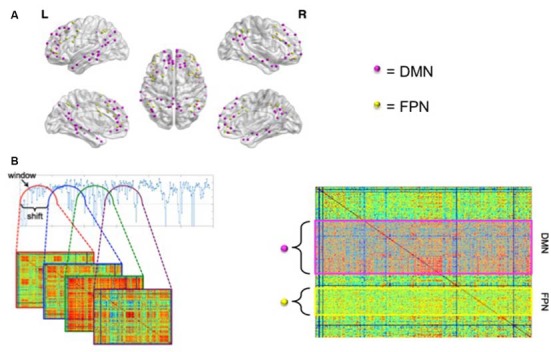
Graphical representation of the calculation of dynamic functional connectivity (dFC). **(A)** Nodes of the default mode network (DMN) are depicted in pink; nodes of the frontoparietal network (FPN) are depicted in yellow. See Power et al. (2011) for specification of the networks. **(B)** A shifting window approach was used to calculate dFC. The left panel shows that an adjacency matrix was calculated for each shifted window. The standard deviation was then calculated for each connection and normalized for its average strength. The right panel of **(B)** shows a matrix of the resulting coefficients of variation for a single subject. Subsequently, an average was calculated for the DMN with the rest of the brain (pink rectangle in right panel) and for the FPN with the rest of the brain (yellow rectangle). Panel **(A)** was visualized using BrainNet Viewer (Xia et al., 2013).

See Figure 1 for a schematic overview of calculation of dFC. Time series of 264 atlas regions were used for subsequent analyses, based on the parcellation suggested by Power et al. (2011). These time series were scrubbed for motion outliers: time points with framewise displacement >1.5 mm (six DOF) were excluded from further analyses. Pearson’s correlation coefficients were calculated between remaining time series from all regions of interest (ROIs) of the Power atlas, resulting in a 264 × 264 matrix per window per subject, with a window length of 60.2 s (28 × TR) and a shift of 10.75 s (5 × TR), resulting in 34 sliding windows per subject. The choice of window length was based on earlier studies (e.g., van Geest et al., 2018). The standard deviation for each connection was calculated and normalized for the average of that individual connection, yielding the coefficient of variation of each connection. This resulted in a separate dFC matrix per individual. Subsequently, we looked at dFC between the regions of the DMN (see Power et al., 2011 for included ROIs) with the rest of the brain, and at dFC between regions of the FPN (see Power et al., 2011 for included ROIs) with the rest of the brain.

### Statistical Analyses

Statistical analyses were performed in IBM SPSS version 23 (Chicago, IL, USA). Normality of all variables was assessed with Kolmogorov-Smirnov tests and histogram inspection. Cognitive test scores were normally distributed, thus independent samples *t*-tests were performed for all cognitive measures, and False Discovery Rate (FDR) correction for multiple comparisons was applied with *Q* < 0.05 (Hochberg and Benjamini, 1995). dFC measures were not normally distributed, thus differences in dFC measures were tested using non-parametric tests (Mann-Whitney). To test the association between dFC and cognition, a hierarchical linear regression using a stepwise forward method was performed per cognitive outcome measure that was significantly different between patients and controls. dFC of the DMN was used as a predictor for memory tests. dFC of the FPN was used as a predictor for tests of executive functioning. Separate hierarchical linear regression analyses were also performed with pain and motor symptoms as dependent variables, and dFC of the DMN and FPN as predictors. Residuals were normally distributed for all regression analyses, justifying the use of these parametric tests. In all regression analyses, average motion during the scan (as a variable) was added in the first step to control for a possible confounding effect of motion despite scrubbing high motion data points. Because of a possible relationship between cognitive decline and dynamics and because differences in static functional connectivity have been found in PD-MCI has been found before on measures of static functional connectivity (Baggio et al., 2015), we divided the group of patients into those with MCI and those without MCI, based on patients’ score on the MoCA. We applied a cutoff score of ≤25 as indicative of MCI (Hoops et al., 2009). Considering the explorative nature of this study, the association between PD symptoms and dFC was tested with an alpha level set at 0.05, without correcting for multiple comparisons.

## Data Availability

The datasets generated and analyzed during the current study are available from the corresponding author on reasonable request.

## Results

### Subjects

**Figure 2:**
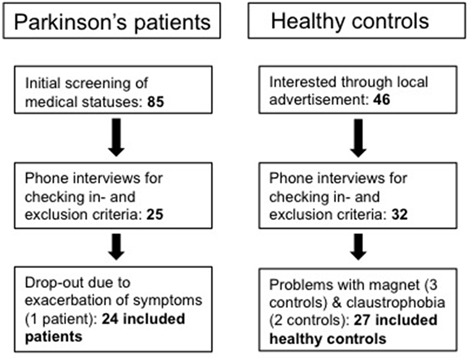
Overview of in- and exclusion process for subjects.

Twenty-four patients and 27 healthy controls participated. See Figure 2 for a flowchart of in- and exclusion of patients. Characteristics of all subjects are shown in Table 1. Patients had a lower level of education than healthy controls (*U* = 174.50, *p* = 0.003). Though not significantly different, patients had a lower score on the MoCA (*t*_(49)_ = 1.9, *p* = 0.063). Patients had a higher score on the BDI, indicating more severe symptoms of depression in patients than in controls (*t*_(48)_ = −5.107, *p* < 0.01).

**Table 1:** Subject characteristics.

	Controls (*n* = 27)	Patients (*n* = 24)	Statistics
Age in years (*M*, *SD*)	59.37 (8.54)	63.42 (7.93)	*t*_(49)_ = −1.749,
			*p* = 0.087
Education level according	6 (3)	5 (4)	*U* = 174.50,
to the Verhage-system			*p* = 0.003
(*Mdn*, range)
Sex	11 females	7 females	$\chi_{\left(1\right)}^{2}$ = 0.745,
			*p* = 0.558
Disease duration in years	-	4.08 (3.13)	-
(M, *SD*)
LEDD in mg (*M*, *SD*)*	-	796.29 (616.44)	-
UPDRS during OFF phase	-	21.08 (8.31)	-
(M, SD)
MoCA (*M*, *SD*)	27.89 (1.89)	26.88 (1.92)	*t*_(49)_ = 1.90,
			*p* = 0.063
BDI	22.96 (2.24)	30.46 (7.12)	*t*_(48)_ = −4.938,
			*p* < 0.001

LEDD, Levodopa Equivalent Daily Dose; MoCA, Montreal Cognitive Assessment; BDI, Beck’s Depression Inventory. *Twelve patients received Levodopa monotherapy, all other patients were taking a combination of Levodopa with DA-agonists or MAO-B/COMT- inhibitors.

### Cognitive Performance

Group differences in cognitive performance are summarized in Table 2. Patients performed worse on the CFR, and on both immediate and delayed recall of the AVLT. Fewer words were named in the verbal fluency tests by PD patients when compared to healthy controls. Interference score for the Stroop was similar for PD patients as for controls. No difference was found for number of digits correctly repeated backwards between PD patients and controls. No difference was found on performance on the BADS between PD patients and controls.

**Table 2:** Group differences in cognitive performance.

	Controls *M* (*SD*)	Patients *M* (*SD*)	Statistics	*p*-value
CFR	24.37 (4.98)	18.56 (7.32)	*t*_(45)_ = 3.17	0.003*
AVLT immediate recall	44.48 (11.48)	37.33 (8.28)	*t*_(49)_ = 2.52	0.015*
AVLT delayed recall	9.26 (3.60)	6.88 (2.54)	*t*_(46.72)_ = 2.75	0.008*
Fluency	19.70 (3.11)	16.94 (4.04)	*t*_(49)_ = 2.74	0.008*
BADS rule shift	32.44 (10.00)	37.04 (13.61)	*U* = 273.5	0.402
Digitspan backwards	6.85 (2.10)	6.33 (2.33)	*t*_(49)_ = 0.841	0.405
Stroop (interference)	0.66 (0.10)	0.62 (0.11)	*t*_(49)_ = 1.22	0.227

AVLT, Auditory Verbal Learning Test; BADS, Behavioral Assessment of the Dysexecutive Syndrome; CFR, Complex Figure of Rey. *Significant, corrected using the False Discovery Rate.

### Dynamic Functional Connectivity

No difference was found between controls (*Mdn* = 0.572) and patients (*Mdn* = 0.941) regarding dFC between the DMN and the rest of the brain (*U* = 288.00, *p* = 0.497). No difference was found between controls (*Mdn* = 0.896) and patients (*Mdn* = 0.962) regarding dFC between the FPN and the rest of the brain (*U* = 318.00, *p* = 0.910).

### Association Between Dynamic Functional Connectivity and Cognitive Performance

**Figure 3:**
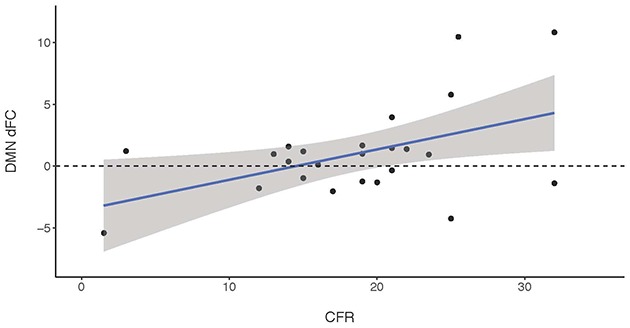
A positive association was found between dFC of the DMN and performance on the visuospatial memory task (Complex Figure of Rey, CFR) in parkinson’s disease (PD) patients.

As described above, performance on the AVLT (both immediate and delayed recall), CFR and verbal fluency was lower in patients compared to controls (see Table 2). With respect to memory functioning, dFC of the DMN was positively associated with performance on the CFR (*R*^2^ = 0.219, *F*_(1, 21)_ = 5.979, *p* = 0.023). dFC of the DMN was not associated with score on the immediate recall, or with score on the delayed recall of the AVLT. With respect to executive functioning, no association was between the dFC of the FPN and performance on verbal fluency tasks. See Table 3 and Figure 3 for details.

**Table 3:** Association between dynamic functional connectivity (dFC) and cognitive functioning.

Network	Cognitive measure	Unstandardized B	Std. error of B	Standardized Beta	*p*-value	Effect size (*R*^2^ change)
DMN	CFR	0.974	0.398	0.502	0.023*	0.219
	AVLT immediate	0.464	0.491	0.212	0.356	0.039
	AVLT delayed	0.214	0.151	0.317	0.171	0.039
FPN	Verbal fluency	0.405	0.267	0.305	0.145	0.083

Linear hierarchical regression analyses were performed with a forward stepwise method, per cognitive outcome measure. DMN, Default Mode Network; FPN, Frontoparietal Network; CFR, Complex Figure of Rey; AVLT, Auditory Verbal Learning Test; *significant association.

Additionally, we investigated dFC differences according to presence of MCI in PD patients. PD patients without MCI had a higher dFC of the DMN with the rest of the brain (*U* = 23.00, *p* = 0.027). There was no difference in dFC of the FPN with the rest of the brain with respect to MCI (*U* = 46.00, *p* = 0.535).

### Association Between Dynamic Connectivity and Other PD Symptoms

A linear hierarchical regression using a forward method was also performed with motor severity and pain intensity as outcome variables. Again, average movement during the scan was added in the first block to control for the possible confounding effect of motion. dFC of the DMN and FPN was not significantly associated with either motor severity or with pain intensity during the OFF phase. See Table 4 for details.

**Table 4:** Association between dFC, pain and motor symptoms.

			Unstandardized B	Std. error of B	Standardized B	*p*-value	Effect size (*R*^2^)
DMN	Motor severity:	UPDRS III	−0.301	0.510	−0.137	0.562	0.016
	Pain experience:	NRS	−0.121	0.140	−0.198	0.398	0.034
FPN	Motor severity:	UPDRS III	0.239	0.250	0.216	0.350	0.042
	Pain experience:	NRS	0.009	0.071	0.031	0.896	0.001

*Linear hierarchical regression analyses were performed with a forward stepwise method, per symptom*.

## Discussion

The main aim of this study was to investigate a link between cognitive performance and dynamics of the DMN and FPN in PD patients. First, we found that both dFC of the DMN and dFC of the FPN with the rest of the brain did not significantly differ between PD patients and controls. In addition, we report dFC of the DMN with the rest of the brain as a correlate of visuospatial memory in our patient group.

The positive association between dFC and visuospatial memory in PD strengthens the findings of a previous study investigating cognitive functioning in PD, also using a sliding window dFC technique: higher dynamics within the dorsal attention network at rest was found to be predictive for attention performance (Madhyastha et al., 2015a). The association between cognition and dFC has also been investigated in other patient populations: in a group of epilepsy patients, lower dFC of the PCC, a key player in the DMN, with the rest of the brain was related to disturbed verbal memory functioning (Douw et al., 2015), suggesting a similar association between dFC and memory functioning as was found in our PD group.

Jones and colleagues (Jones et al., 2012) have suggested before that dFC of the DMN might underlie the cognitive deterioration in people at risk for developing Alzheimer’s disease (AD) patients. Our results established a lower dFC of the DMN with the rest of the brain for patients with MCI. These results hint that Jones’ hypothesis could also hold for PD, and that dFC of the DMN with the rest of the brain is linked to cognitive fitness, or to the preservation of cognitive functioning.

We found no group differences in dFC of either network (i.e., DMN or FPN), even though patients’ cognitive functioning was worse on several tests. This could be due to methodological choices, such as our focus on dFC of the entire FPN and DMN. Consideration of further subdivisions of each network (e.g., posterior DMN vs. anterior DMN) may increase sensitivity to group differences. This is evident in a study of people with autism spectrum disorder, which showed dFC increases in certain subsets of the DMN and decreases in other subsets of the DMN when compared to controls (de Lacy et al., 2017). Another example is the study of Jones and colleagues (Jones et al., 2016), where dwell time in several subnetworks of the DMN was calculated for a group of AD patients, using a sliding window technique for resting-state connectivity. AD patients spent more time in the anterior subnetwork of the DMN, but less time in the posterior subnetwork of the DMN. Thus, focusing on different subnetworks of the DMN might improve specificity of conclusions on group differences.

Our second aim was to test whether dFC was specific for certain aspects of cognitive functioning, or whether it might also be related to motor symptoms or pain experience. No association was found between dFC and motor symptoms, or dFC and pain. This does not rule out the possibility of an association between dFC and other symptoms of PD: in the recent study by Kim et al. (2017), severity of motor symptoms was related to time spent in a specific configuration of sparse interconnectedness, which was calculated using a sliding window technique. However, our results suggest that cognition is the main correlate of dFC of the DMN specifically. To our knowledge, however, no study has investigated a link between dFC and pain in PD. More research is needed to investigate to what extent dFC might be specifically underlying cognitive functioning as opposed to other symptoms of PD. Future studies could, for example, select patients on motor-subtypes, such as tremor, akinesia, rigidity or loss of balance.

The results of our study indicate that the dFC of the DMN is related to visuospatial memory in PD patients. This association between dFC and cognitive functioning is not only interesting from a clinical point of view, but it also strengthens the notion that dynamic connectivity is linked to brain function (Hutchison et al., 2013; Sizemore and Bassett, 2017). However, some limitations apply. First, the number of subjects included in this study is small, and so results cannot be generalized to the entire population of PD patients. Given the heterogeneity of symptoms and symptom severity in the population of PD patients together with the relatively mild symptoms present in our small sample, one should be careful in extrapolating these results to the entire population of PD patients. Second, factors such as long-term effects of medication and disease duration were not taken into account. Third, cognitive functioning was tested in the ON phase, whereas resting-state data came from the OFF phase. A stronger link might be found if neuropsychological testing would occur in the same medication state as the resting-state scan. Fourth, one should take into account that dynamic connectivity has been introduced quite recently, and its biological correlates are not yet firmly established. Additionally, we report only a correlation between dynamics and cognition in this study. Longitudinal studies may elucidate whether a decrease of dFC precedes cognitive deterioration in PD or vice versa. Animal and intervention studies, such as pharmacological and brain stimulation studies, could provide evidence for a causal link between brain dynamics and cognitive functions, and determine its exact implications.

## Conclusion

This cross-sectional study reports dFC as a neural correlate of cognitive functioning in PD: dFC of the DMN with the rest of the brain was associated with better visuospatial memory functioning. This association was not found when motor symptoms or pain were considered, which suggests that dFC of the DMN may be specifically linked to cognitive functioning. This study adds to the understanding of which factors possibly contribute to cognitive functioning in PD.

## Author Contributions

GE, BM, AV and ES conceived and designed the experiments. GE and BM performed the experiments. GE, BM and LD analyzed the data. GE, BM, AV, ES and LD wrote the article.

## Conflict of Interest Statement

The authors declare that the research was conducted in the absence of any commercial or financial relationships that could be construed as a potential conflict of interest.
